# Progressive Provisioning by the Females of the Earwig, *Anisolabis maritima*, Increases the Survival Rate of the Young

**DOI:** 10.1673/031.010.14144

**Published:** 2010-10-22

**Authors:** Seizi Suzuki

**Affiliations:** Center for e-Learning Research and Application, Nagaoka University of Technology 1603-1 Kamitomioka, Nagaoka, Niigata 940-2188, Japan

**Keywords:** defending behavior, food provisioning, maternal care

## Abstract

Provisioning the young is an important form of insect parental care and is believed to improve the survival and growth of the young. *Anisolabis maritima* Bonelli (Dermaptera: Anisolabididae) is a cosmopolitan species of earwig that shows sub-social behavior in which the females tend clutches of eggs in soil burrows. The defensive and provisioning behaviors of these females were examined in this study. When disturbed, maternal individuals abandoned the nest less than non-maternal individuals. Females brought food to the nest after their eggs hatched, and the survival of the nymphs was increased by provisioning. Even when mothers were removed, providing food to the nymphs increased survival as well as when the nymphs were provisioned by the mother. These results show that *A. maritima* mothers provision the nymphs and that this provisioning improves their survival.

## Introduction

Parental care has evolved considerably across several taxa of animals (Clutton-Brock 1991). In insects, parental care is not common, but it is known in different lineages. Several insect species have developed parental care that varies in its form and degree of sociality (Tallamy and Wood 1986; Costa 2006). Guarding of eggs and early-stage nymphs is the type of parental care most frequently observed, and is believed to have evolved in response to intense arthropod predation pressure (Costa 2006). Provisioning, an advanced form of parental care, has been reported for several insect species (Scott 1998), and progressive provisioning, parents repeatedly transporting food to their young, has also been reported (Filippi-Tsukamoto et al. 1995; Filippi et al. 2001). Although progressive provisioning would enhance the survival of young, there have been few reports on species showing progressive provisioning other than in Hymenoptera and Isoptera.

All earwig (Dermaptera) species studied to date exhibit parental care (Lamb 1976), however, the extent of care varies greatly from species to species (Vancassel 1984). For example, *Tagalina papua* shows only egg guarding (Matzke and Klass 2005), but the hump earwig, *Anechura harmandi,* mothers guard and clean the eggs and are killed and eaten by the first-instar nymphs before they disperse from the nest (Kohno 1997; Suzuki et al. 2005). Although nearly 2,000 Dermaptera species have been described (Haas 2003), parental behavior has been examined in only a handful of these species. Furthermore, the mothers of some species have been reported to provision their nymphs (Shepard et al. 1973; Lamb 1976; Rankin et al. 1996; Kölliker 2007), but the effect of provisioning on the survival of the nymphs remains unknown.

*Anisolabis maritima* Bonelli (Dermaptera: Anisolabididae) is a cosmopolitan species that shows sub-social behavior in which the females tend clutches of eggs in soil burrows (Bennett 1904). Mothers of this species bring food to the nest (Guppy 1950). The present study examined the maternal behavior of *A. maritima* and focused on whether mothers provision their nymphs progressively and whether provisioning improves the survival of the nymphs.

## Materials and Methods

All *A. maritima* individuals were caught in a field on the coast of Izumozaki, Niigata Prefecture, Japan (138° 42′ 10″ N, 37° 32′ 11″ E) between late April and early May in 2008 and 2009. All females were coupled with a male for 1–2 days prior to the start of the experiment. After body length was measured, the females were placed together in a polyethylene container (12 × 8 × 5 cm) with some sand and a small stone as shelter. The containers were maintained under dim light conditions, at room temperature, and under sufficient humidity. All individuals were fed turtle food pellets *ad libitum.* All containers holding a female with an egg mass were checked daily. When hatched nymphs were found, the containers were assigned to an experiment.

### Observation of defending behavior

Some females before first oviposition were assigned as non-caring females. Both noncaring (*n* = 20) and caring (attending nymphs, *n* = 24) females were approached from the front and gently touched on the back with forceps three times. The initial responses shown by the females during these disturbances were recorded.

### Observation of provisioning behavior

Bottle caps (25 mm in diameter, 10 mm in depth) placed at a distance of 2–3 cm from the burrow were used as food containers. Immature (before dispersal) nymphs were not able to enter the bottle cap to eat (S Suzuki, personal observation). The food provided was 10 turtle food pellets (average 0.07 g total). Each container (*n* = 16) was checked daily; the number of remaining pellets were counted, and pellets were added as necessary to again keep a total of 10. When more than half of the nymphs left the nest, or some nymphs were found in the bottle cap, the brood was considered to have dispersed.

### Effects of provisioning on nymph survival

All containers holding a female with an egg mass were checked daily, and when hatched nymphs were found, the containers were assigned randomly to an experiment. In the mother-removal group (*n* = 14), mothers were removed just after hatching and their nymphs were maintained without food. In the feeding group (*n* = 13), mothers were removed, and 10 food pellets per day were provided in the nests as food. The leftover food was replaced every day. In the non-feeding group (*n* = 14), nymphs were maintained with the mother but no food was provided. In the control group (*n* = 15), nymphs were maintained with the mother and 10 food pellets per day were provided in bottle caps to allow provisioning by the mother. After eight days, the number of surviving nymphs was counted.

**Table 1.**
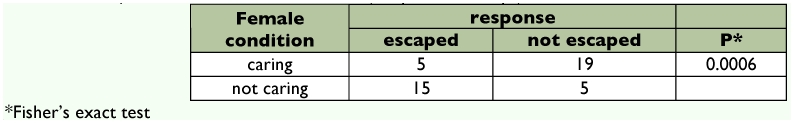
Response of females to disturbance (3 taps with forceps).

## Results

### Observation of defending behavior

When disturbed by forceps, the females showed three different response types: (1) remaining immobile, (2) counterattacking, or (3) running away. When a female ran away from its initial position before the three taps with the forceps were completed, it was recorded as “escaped.” Since caring females without disturbance always stay in the nest or cover their nymphs, remaining immobile can be regarded as a defensive behavior. Fifteen out of 20 females not attending nymphs escaped after three taps, but 19 out of 24 females attending nymphs did not (P=0.0006, Fisher's exact test, Table 1). Counterattacks were observed in 3 cases of females attending nymphs.

### Observation of provisioning behavior

**Figure 1.**
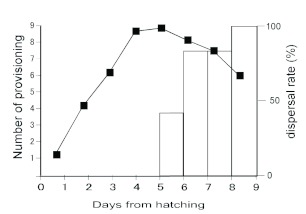
Daily changes in the average number of instances of food provisioning (line plot) and dispersal rate (bar chart). High quality figures are available online.

The females did not carry any food until after hatching, when they began to carry food with their mouth to the nest. The number of instances of food carrying and dispersal days are shown in Figure 1.

Nymphs dispersed from the nest in 5.9 ± 0.9 days (mean ± SD). There was leftover food in most nests, though the pellets were crumbled and could not be counted. Mouthpart-to-mouthpart contact was not observed between mothers and nymphs, and the nymphs ate the food themselves in the nest.

### Effects of provisioning on nymph survival

**Figure 2.**
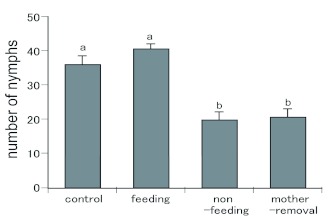
Effects of the presence of the mother and food provisioning on brood survival (mean ± SE). Bars with different letters are significantly different (Tukey-Kramer method, p < 0.05). High quality figures are available online.

Fewer nymphs were observed in the broods in both the mother-removal and non-feeding groups than in the control and feeding groups (Figure 2, F = 20.3, d.f = 3, p < 0.01, TukeyKramer method).

## Discussion

Nesting and brood attendance are found throughout the order Dermaptera (Costa 2006). Previous studies have reported food provisioning to nymphs by earwig parents in some species, based on observations of mouthpart-to-mouthpart contact (Lamb 1976) and direct evidence (Staerkle and Kölliker 2008). However, to the author's knowledge, no research has been conducted to examine the effect of provisioning on the survival of the nymphs. The results of the present experiments demonstrate that when mothers are removed, fewer nymphs survive. When food was provided to the nymphs whose mother was removed, however, they survived as well as when the mother was present. In contrast, fewer nymphs survived under the mother-removal treatment (no food). Although the mortality factor was not directly observed, the absence of dead nymphs in the mother-removal treatment suggests sibling cannibalism. Food con-sumption could be quantified only on the family group level, confounding food provisioning and larval/female self-feeding. Larval feeding and self-feeding by mother could not be distinguished in this study. However, since nymphs before dispersal cannot enter a bottle cap to eat, this result is direct evidence of food provisioning by the mother. These results indicate that food being provided for the hatched nymphs is a prerequisite for their survival.

Progressive provisioning is well known in organisms from higher taxa such as birds and mammals (Clutton-Brock 1991) and is essential for the survival of young in these altricial species. In contrast, reports of progressive provisioning are rare among insects other than species in Hymenoptera and Isoptera. For example, the burrower bug, *Parastrachia* spp., feed their nymphs fallen drupes (Filippi et al. 2001; Kölliker et al. 2006), and crickets (*Anuro gryllus* spp.) breed in underground burrows to which the mother brings food for the young (Walker 1983). Since *A. maritima* females brought food back to the nest for several days (Figure 1), this behavior can be regarded as progressive provisioning. In another Dermaptera species, *Forficula auricularia,* females regurgitate food (Staerkle and Kölliker 2008), but the nymphs of *A. maritima* were not observed in any mouthpart-to-mouthpart contact in the present study. Since *A. maritima* mothers provision food only by placing it in the nest, food allocations to individual nymphs have not been confirmed. However, since the mothers provision increasing amounts of food with increasing days from hatching (Figure 1), they could control food mass according to the needs of the nymphs.

The results of the feeding group indicate the self-feeding ability of the nymphs. Even in the European earwig, which is reported to be fed directly by the mother (Staerkle and Kölliker 2008), nymphs are able to feed directly, which is important to survival (Kölliker 2007). The present study was conducted under no predation risk and with artificial food. In cases where there may be no food for the nymphs in the nest, the nymphs must leave the nest to feed without provisioning by the mother. Sub-social insect species showing progressive provisioning often face a high predation risk of nymphs (Filippi-Tsukamoto et al. 1995). Earwig nests also suffer high predation pressure from various animals (Kohno 1997; Kölliker and Vancassel 2007). Many females attending nymphs stayed in the nest even when disturbed by the forceps (Table 1). Since this can be regarded as defensive behavior, reduced predation pressure is expected with maternal attendance. Nymphs will suffer from predation pressure and starvation without mother attendance. Filippi et al. (2000) demonstrated that in the sub-social shield bug, *Parastrachia japonensis,* progressive provisioning enhances nymphal survival in high predation-pressure environments by inhibiting nymphal dispersal from safe nesting sites. Staying in the nest decreases the risk of predation, and provisioning by the mother decreases the risk of starvation.

It is difficult to distinguish provisioning from young/female self-feeding. The present study confirmed provisioning by *A. maritima* females by providing food using a barrier that nymphs can not cross and showing an improved survival rate in the presence of food. This provides evidence in favor of the effectiveness of progressive provisioning and defensive behavior by *A. maritima* mothers under laboratory conditions. Food provisioning was the primary aspect of care that influenced the benefits of maternal attendance in the present study.

## References

[bibr01] Bennett C (1904). Earwigs (*Anisolabis maritima* Bon.).. *Psyche*.

[bibr02] Clutton-Brock TH (1991). *The Evolution of Parental Care.*.

[bibr03] Costa JT (2006). *The Other Insect Societies.*.

[bibr04] Filippi L, Hironaka M, Nomakuchi S (2001). A review of the ecological parameters and implications of subsociality in *Parastrachia japonensis* (Hemiptera: Cydnidae), a semelparous species that specializes on a poor resource.. *Population Ecology*.

[bibr05] Filippi L, Hironaka M, Nomakuchi S, Tojo S (2000). Provisioned *Parastrachia japonensis* (Hemiptera: Cydnidae) nymphs gain access to food and protection from predators.. *Animal Behaviour*.

[bibr06] Filippi-Tsukamoto L, Nomakuchi S, Kuki K, Tojo S (1995). Adaptiveness of parental care in *Parastrachia japonensis* (Hemiptera: Cydnidae).. *Annals of the Entomological Society of America*.

[bibr07] Guppy R (1950). Biology of *Anisolabis maritima* (Gene) the seaside earwig, on Vancouver Island (Dermaptera, Labiduridae).. *Proceedings of the Royal Entomological Society of British Colombia*.

[bibr08] Haas F, Dathe FF (2003). Ordnung Dermaptera Ohrwürmer.. *Lehrbuch der Speziellen Zoologie, Band 1: Wirbellose Tiere, 5. Teil: Insecta*.

[bibr09] Kohno K (1997). Possible influences of habitat characteristics on the evolution of semelparity and cannibalism in the hump earwig *Anechura harmandi*.. *Researches on Population Ecology*.

[bibr10] Kölliker M (2007). Benefits and costs of earwig (*Forficula auricularia*) family life.. *Behavioural Ecology & Sociobiology*.

[bibr11] Kölliker M, Chuckalovcak JP, Haynes KF, Brodie ED (2006). Maternal food provisioning in relation to condition-dependent offspring odours in burrower bugs (*Sehirus cinctus*).. *Proceedings of the Royal Society of London*, Series B.

[bibr12] Kölliker M, Vancassel M (2007). Maternal attendance and the maintenance of family groups in common earwigs (*Forficula auricularia*): A field experiment.. *Ecological Entomology*.

[bibr13] Lamb RJ (1976). Parental behavior in the Dermaptera with special reference to *Forficula auricularia* (Dermaptera: Forficulidae).. *Canadian Entomologist*.

[bibr14] Matzke D, Klass K-D (2005). Reproductive biology and nymphal development in the basal earwig *Tagalina papua* (Insecta: Dermaptera: Pygidicranidae), with a comparison of brood care in Dermaptera and Embioptera.. *Entomologiche Abhandlungen*.

[bibr15] Rankin SM, Storm SK, Pieto DL, Risser AL (1996). Maternal behavior and clutch manipulation in the ring-legged earwig (Dermaptera: Carcinophoridae).. *Journal of Insect Behavior*.

[bibr16] Shepard M, Waddil V, Kloft W (1973). Biology of the predaceous earwig *Labidura riparia* (Dermaptera: Labiduridae).. *Annals of the Entomological Society of America*.

[bibr17] Staerkle M, Kölliker M (2008). Maternal food regurgitation to nymphs in earwigs (*Forficula auricularia*).. *Ethology*.

[bibr18] Scott MP (1998). The ecology and behavior of burying beetles.. *Annual Review of Entomology*.

[bibr19] Suzuki S, Kitamura M, Matsubayashi K (2005). Matriphagy in the hump earwig, *Anechura harmandi* (Dermaptera: Forficulidae), increases the survival rates of the offspring.. *Journal of Ethology*.

[bibr20] Tallamy DW, Wood TK (1986). Convergence patterns in subsocial insects.. *Annual Review of Entomology*.

[bibr21] Vancassel M (1984). Plasticity and adaptive radiation of Dermapteran parental behavior: Results and perspective.. *Advances in the Study of Behavior*.

[bibr22] Walker TJ, Gwynne DT, Morris GK (1983). Mating modes and female choice in short-tailed crickets (*Anurogryllus arboreus*).. *Orthopteran Mating Systems: Sexual Competition in a Diverse Group of Insects.*.

